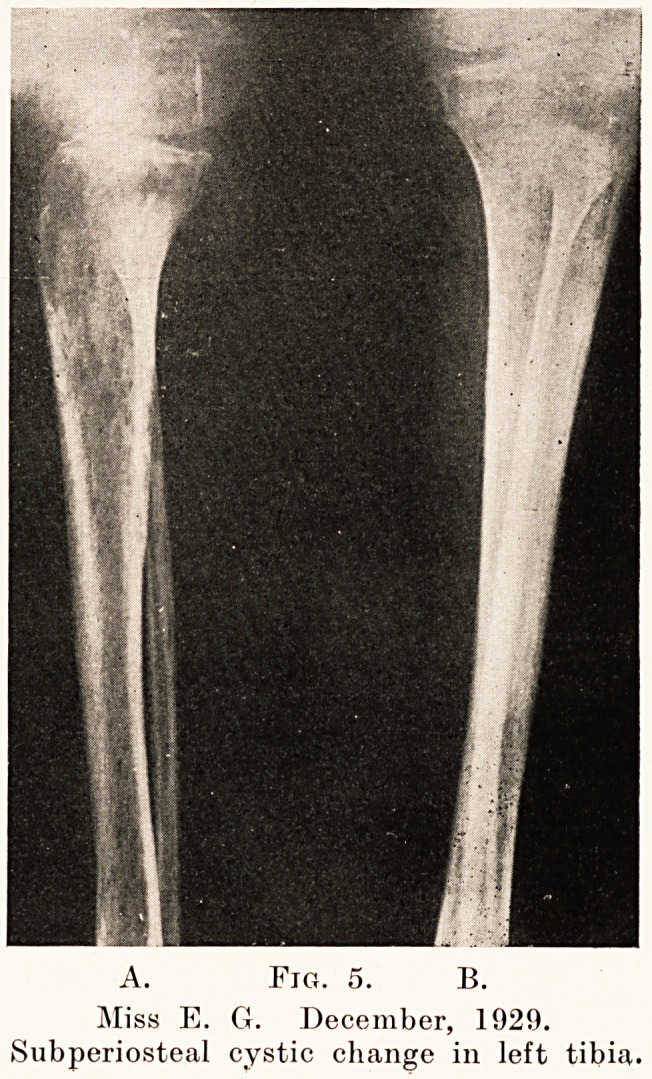# A Case of Von Recklinghausen's Disease (Multiple Neurofibromata) with Spontaneous Fractures
*This Essay was awarded a Martyn Memorial Pathological Scholarship.


**Published:** 1930

**Authors:** L. P. Ashton


### A CASE OF VON RECKLINGHAUSEN'S DISEASE (MULTIPLE NEUROFIBROMATA) WITH SPONTANEOUS FRACTURES.*

BY

L. P. Ashton.

---

The case to be described was one in which spontaneous fractures of the neck of each femur occurred, the skiagrams showing well-marked rarefaction in the ends of these bones.

The condition as originally described by von Recklinghausen was that of changes in the skin and cutaneous nerves alone; further research on the subject, however, has disclosed bone changes among the disturbances associated with the disease.

Although von Recklinghausen, in 1882, was the first to describe the pathology of the disease, it is interesting to note that one of our own countrymen, Robert W. Smith, after research in two cases, published an account of the condition in 1849. Von Recklinghausen, however, was the first to offer the now accepted theory of neuro-fibromatosis.

The disease is characterized by multiple painless nodules in the skin, irregularly distributed over the whole trunk and limbs, usually most abundant on the abdomen and back. The nodules are situated in the skin or subcutaneous tissues, and are not usually adherent to deeper structures. They may become

* This Essay was awarded a Martyn Memorial Pathological Scholarship.

pedunculated, but often remain deep enough to be invisible on the surface. They vary in size from a few millimetres to an inch or more in diameter; and may even develop to an enormous size when they become pedunculated, causing the condition of elephantiasis neuromatosa.

Microscopical examination reveals a fibrous nodule arising in a cutaneous nerve filament. It may be on the side or in the centre of a nerve, and in the latter case the nerve fibres will be seen spreading out and enclosing the tumour. This is said to originate from the connective tissue elements of the nerves, especially from the sheath of Schwann; tumours never arise in the olfactory or optic nerves, which are devoid of this sheath. The structure presents interlacing hyaline fibres, between which lie a few or many compressed tumour cells, the older tumours being almost completely fibrous. All the elements of the nerve filament or nerve trunk, except the ganglion cells and nerve fibres, participate in the tumour process. Nerve fibres in a state of degeneration can usually be demonstrated passing either over or through the tumour. Later in the disease the nerve trunks are similarly affected in any situation between the nerve root and the periphery. Motor or sensory phenomena follow damage to these nerves from pressure, especially if the tumour arises near the nerve root in the spinal canal or cranium.

Pigmentation is present in most cases in the form of freckles, blotches, or diffuse areas, often situated in relation to the underlying nodule.

*Bone changes* are said to occur in about 7 per cent. of the cases. There is some diversity of opinion as to the exact nature of the change, but the weight of evidence goes to show that the lesion is similar to that of osteomalacia. There can be little doubt that the condition is not in any way connected with osteitis fibrosa, for as Gould[1] demonstrated, there was no deposition of fibrous tissue in the medullary cavity of a femur examined by him, a feature always present in osteitis fibrosa.

In research carried out on microscopical sections of bones in von Recklinghausen's disease Gould showed that, in a decalcified bone, there was absence of abnormality in general structure and in the relative proportions of bone substance and marrow. The Haversian spaces were quite normal and were nowhere dilated, the condition found in very advanced osteomalacia.

In an undecalcified section the main feature was the large amount of bone devoid of lime salts, that is in the condition of osteoid tissue. The centrally-placed bone could be seen surrounded by the osteoid tissue, which also lined the Haversian canals and covered the bony trabeculae. The osteoid present was considerably in excess of the true bone. These changes are similar to those seen in early osteomalacia, and, consequently, suggest the connection between that condition and the disease under consideration. Both conditions are possibly brought about by alteration of internal secretions, the manifestations of both being exaggerated by pregnancy, becoming less again after delivery.

Subperiosteal nodules are frequently present, and on section there have been found to be swellings covered by very cellular periosteum and containing large irregular bone corpuscles. Brooks and Lehman[2] state that subperiosteal nerve involvement leads to the reaction of destruction and regeneration of bone; a thin shell of bone is deposited by the periosteum which may be palpated on the bone. In consequence of the destructive process cysts are formed under the periosteum; these may be small (as in the left tibia, Fig. 5, a), or may reach the size of the thickness of the bone. These cysts usually become filled with dense fibrous tissue. The hyperæmia brought about by the reaction throughout the bone may cause thickening and lengthening of the bone affected.

The disease may be hereditary or congenital. Osler states that in one family three generations were affected, and in another the mother and several children. On the other hand, the bone changes and pigmentation possibly suggest some endocrine disturbance as the underlying cause. No definite conclusion, however, has yet been reached.

The clinical record of the case is as follows:—

Female, aged 47. Domestic servant. She gave a history of erythema nodosum in 1924 and broncho-pneumonia with pleural effusion in 1927. In the family history no traces of von Recklinghausen's disease can be found.

*Present history.*—The patient was taken for the first time with sudden sharp pain in the right hip-joint, causing her to fall down. After admission to the Bristol General Hospital an X-ray examination revealed a transverse fracture to the neck of the left femur. Eleven months later, after the patient had been walking round the ward with the aid of crutches and Thomas's walking calliper, another skiagram was taken, which showed a fracture of the neck of the right femur, the lesion having occurred without previous signs or symptoms.

*State on Examination.*—The patient was thin and pale with a dry, rough skin. A number of small painless nodules up to 1 c.m. in diameter were present on the trunk, some rounded, others pedunculated. Blotchy pigmentation was present on the abdomen and chest, and in the form of freckles on the legs and arms.

The flexures of the limbs were the sites of firm, nodular enlargements, which were deep to the tendons but not fixed to the bone, and did not appear in the skiagrams. These swellings were seen at the elbows, wrists, knees, and groins,

### PLATE XIII.

Fig. 1.
Miss E. G. December, 1929.
X-ray to show changes in ribs and right humerus.

Fig. 2.
Miss E. G. December, 1929.
X-ray to show change in shaft of left femur.

Fig. 3.
Miss E. G. December, 1929.
Showing fracture of the necks of both femora.

### PLATE XIV.

FIG. 4.
Miss E. G. December, 1929.
Advanced rarefaction near left knee-joint.

A. FIG. 5. B.
Miss E. G. December, 1929.
Subperiosteal cystic change in left tibia.

and the average size was equivalent to that of a walnut. Growth was very slow except in the case of the one in the right groin, which attained the size of a tennis ball in a few weeks ; this tumour was smooth, round and fixed, and the femoral artery could be seen pulsating superficially to it. None of these swellings gave rise to pain, and there was no history of previous neuralgia or headache. After a year no further nodules appeared, and the ones already present decreased considerably in size. Subperiosteal nodules could be palpated along the posterior border of each ulna and down the antero-internal surface of each tibia.

A nodule was excised from the anterior fold of the left axilla and sections were cut. Microscopically the section consisted of fibrous tissue with only a few scattered tumour cells between the fibres, indicating that the tumour was one of somewhat slow growth.

*Bone Changes.*—The entire skeleton was X-rayed twice with an interval of nine months between. The change in the bone appeared to be progressing slowly in the tibia and left femur, and to be stationary in the remainder of the skeleton.

The skiagram taken in December, 1929, showed fractures in the neck of each femur, with very advanced absorption of bone in the region of the fracture on the left side, the neck being almost completely absorbed on that side. (Fig. 2.)

Three types of rarefaction could be demonstrated in the skiagrams :—

1. A diffuse type was seen in the pubes, lumbar vertebræ and thorax, probably due to disuse. (Fig. 3.)

2. A patchy type was well marked in the left femur and tibia. This change was seen mostly in the neighbourhood of joints, those affected to the greatest extent being the knee and ankle, but similar changes were also present near the elbow, wrist and hand joints.

This discrete patchy type appeared to be due to the subperiosteal nodules and cysts which have been demonstrated in the bone. These appeared in the skiagram, and could be palpated in the respective bones, *i.e.* tibia and ulna, one situated on the tibial crest being most readily demonstrated.

3. A somewhat grosser cystic type of rarefaction with the diffuse type superimposed was present in the proximal two-thirds of each femur and humerus, also in the patella and condyles of the left femur. (Figs. 1 and 3.) It was in these areas that the osteomalacic changes were most marked, whereas in the left tibia the subperiosteal nodules predominated.

Types 2 and 3 were clearly the result, not of simple disuse, but of definite neurotrophic disturbance of a different character.

*The Joints.*—There was much thickening of peri-articular tissue in the ring and little finger of each hand, causing complete immobility of these fingers. The other fingers were affected, but to a lesser degree. The swellings in the flexures of the other joints described above were the cause of limited movement, the joints themselves after careful examination appearing to be quite normal.

The only nerve change that could be demonstrated was the complete loss of pain, temperature and touch sense in the index and middle fingers of the left hand. This had been present for many years. This lack of nerve signs went to show that there was little or no involvement of nerve roots in the spinal canal and cranium.

In reference to the calcium deficiency of the bone, the blood calcium was estimated and found to be slightly reduced—0·0092 per cent., the normal being 0·01 per cent. This was not enough to account for the excessive loss of calcium in the bone.

To exclude specific changes in the hip-joints the Wassermann reaction was carried out and found to be negative.

### SUMMARY.

A case of von Recklinghausen's disease (multiple neurofibromata) is described in which bone changes were noted. These included spontaneous fracture of the necks of both femora. The nature of these bone changes is discussed.

Thanks are due to Dr. Carey Coombs for permission to use the notes and to Dr. Macdonald Critchley for his comments on the case.

### REFERENCES.

[1] Pearce Gould, *Quarterly Journal of Medicine*, Oxford, 1918, x. 24.

[2] Brooks and Lehman, *Surg. Gyn. and Obs.*, Chicago, 1924, xxxviii. 587–595.

## Figures and Tables

**Fig. 1. f1:**
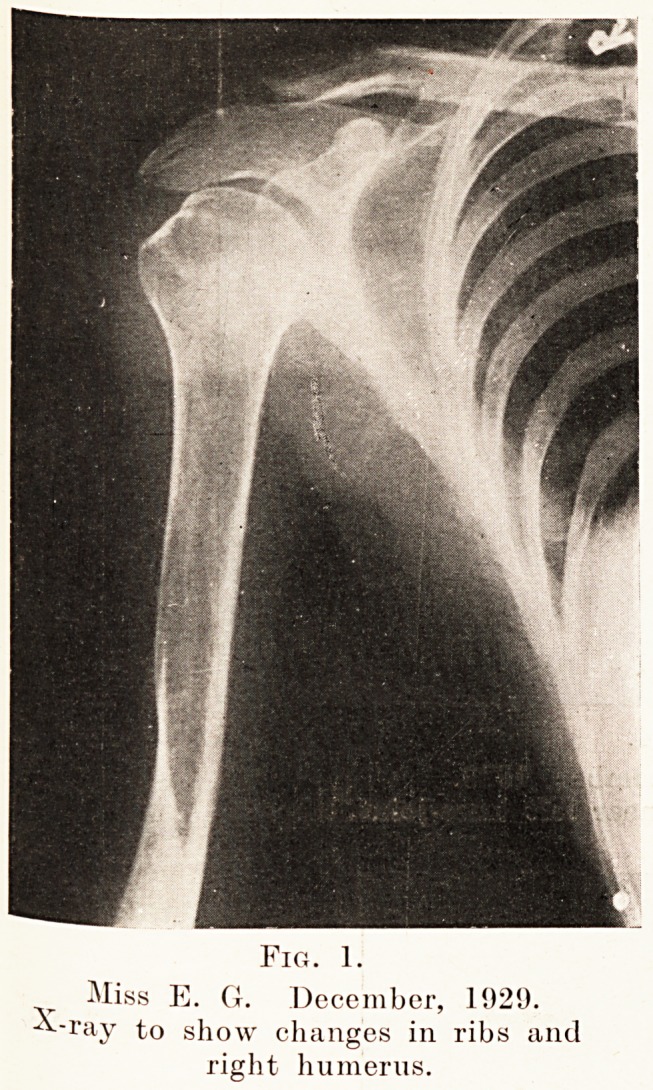


**Fig. 2. f2:**
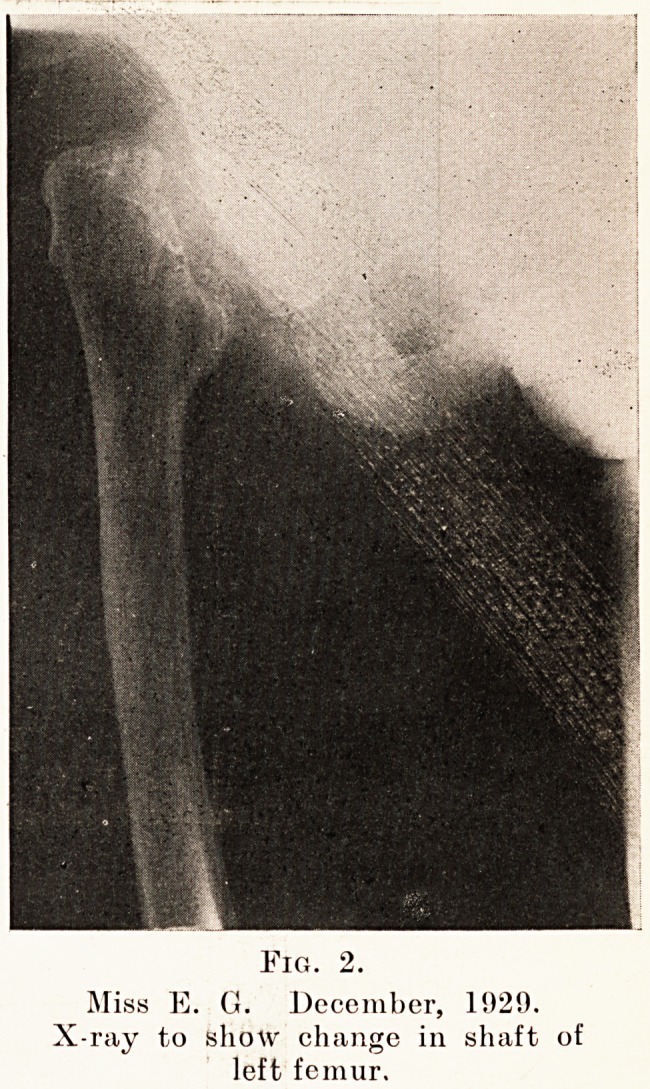


**Fig. 3. f3:**
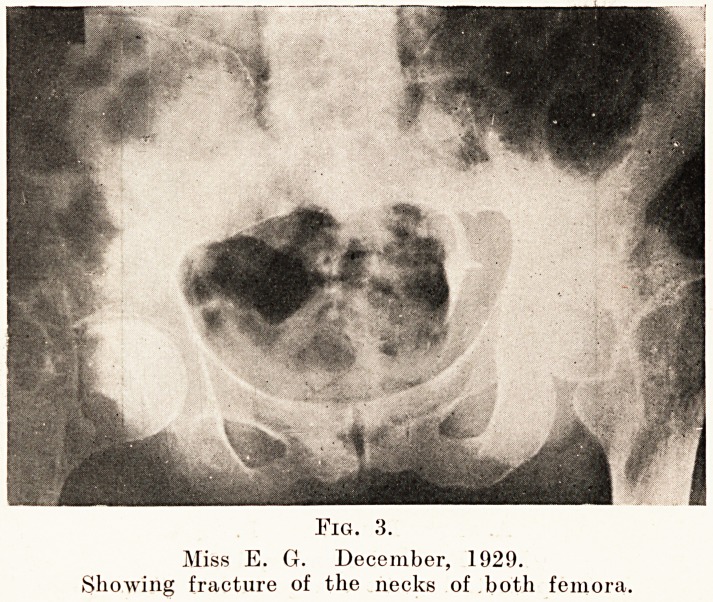


**Fig. 4. f4:**
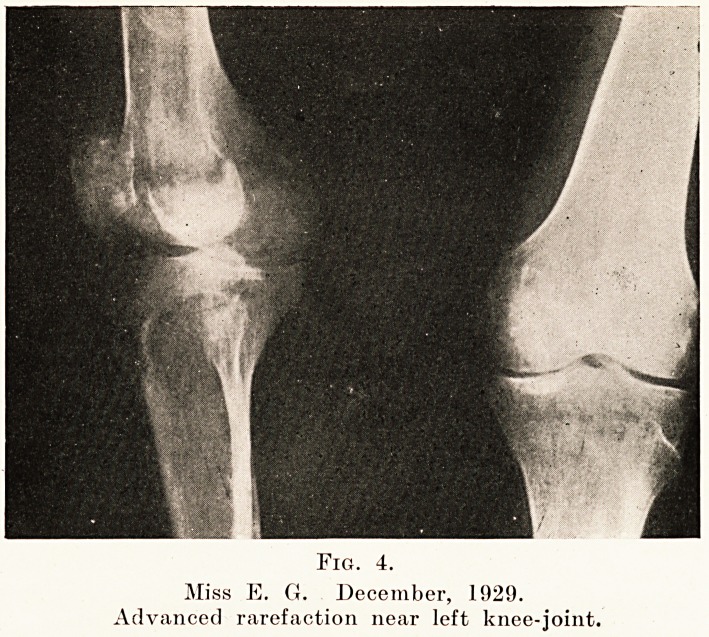


**Fig. 5. f5:**